# Soybean Callus—A Potential Source of Tocopherols

**DOI:** 10.3390/plants10122571

**Published:** 2021-11-25

**Authors:** Liliana Mureșan, Doina Clapa, Teodor Rusu, Thomas T. Y. Wang, Jae B. Park

**Affiliations:** 1 Diet, Genomics and Immunology Laboratory, Beltsville Human Nutrition Research Center, Agricultural Research Service, U.S. Department of Agriculture, Beltsville, MD 20705, USA; Liliana.Muresan@montgomerycollege.edu (L.M.); tom.wang@usda.gov (T.T.Y.W.); jae.park@ars.usda.gov (J.B.P.); 2 Institute of Advanced Horticulture Research of Transylvania, University of Agricultural Sciences and Veterinary Medicine Cluj-Napoca, Mănăștur 3-5, 400372 Cluj-Napoca, Romania; 3 Faculty of Agriculture, University of Agricultural Sciences and Veterinary Medicine, Mănăștur St. 3-5, 400372 Cluj-Napoca, Romania; trusu@usamvcluj.ro

**Keywords:** *Glycine max*, in vitro culture, α tocopherol, γ tocopherol, δ tocopherol, HPLC

## Abstract

In vitro cultures have been used as an effective means to achieve a high level of secondary metabolites in various plants, including soy. In this study, the contents of α-, γ-, and δ- tocopherol were quantified in soybean callus, and their amounts were compared to those of soybeans cultivated using the conventional tillage system with three weed controls (respectively without herbicide and with two variants of herbicide). Soybean callus was produced using Murashige and Skoog 1962 (MS) medium supplemented with 0.1 mg/L 6-Benzylaminopurine (BAP) and 0. 1 mg/L Thidiazuron (TDZ). The highest amount of fresh callus was obtained from soybeans from the conventional tillage system with second weed control (S-metolachlor 960 g/L, imazamox 40 g/L, and propaquizafop 100 g/L) respectively 13,652.4 ± 1177.62 mg. The analyzed tocopherols were in much higher content in soy dry callus than the soybean seeds (5.63 µg/g compared with the 0.35 α-toco in soybean, 47.57 µg/g compared with 18.71 µg/g γ-toco or, 5.56 µg/g compared with 1.74 µg/g β-toco). The highest content of the three analyzed tocopherols was γ -tocopherol, both in callus and soybeans. Furthermore, the data showed that herbicides used in soybean culture significantly influenced both the in vitro callus production and the tocopherol callus content (*p* ˂ 0.05). Altogether, soybean callus can be an important source of tocopherols, and herbicides significantly influence in vitro callus production and the tocopherol callus content.

## 1. Introduction

Soybean (*Glycine max* (L.) Merr.) is a key species used by the nutraceutical and functional food industries for its secondary metabolites such as polyphenolic compounds, tocopherols, tannins, lignins, and alkaloids. Particularly, soybean is an excellent source of tocopherols with about 300 μg g^−1^/seed total tocopherols [1]. In fact, tocopherols exist in four forms (i.e., α, β, γ, and δ). Among them, γ-tocopherol is found in higher concentrations (more than 60%), and α-tocopherol accounts for less than 10% of total tocopherols in soybean seeds [2]. Therefore, as the α-tocopherol is an essential nutrient that functions as a peroxyl radical scavenger in the body, the dietary reference intake for vitamin E is currently based solely on α-tocopherol [3,4]. Despite the lesser presence, α-tocopherol is reported to have the most significant antioxidant activity among tocopherols and is preferred by the human body, as it is the predominant form of vitamin E found in the blood and tissues. α-tocopherol is a fat-soluble antioxidant and serves as a peroxyl radical scavenger protecting the polyunsaturated fatty acids in membranes and lipoproteins (protects the fats in low-density lipoproteins from oxidation) [5]. Furthermore, vitamin E is of vital importance for the nervous system, as a deficiency in humans leads to ataxia and myopathy [6]. α-tocopherol is found naturally in plant sources as RRR-a-tocopherol and synthetically manufactured as all-rac-a-tocopherol.

The synthetic all-rac-a-tocopherol consists of a mixture of all eight possible stereoisomers, making the natural vitamin E not equivalent with its synthetical form as their molecular structure is not identical. Furthermore, the bioavailability of natural RRR- is higher than synthetic all-rac-a-tocopheryl with a 2:1 ratio (of RRR: all-rac) [7]. Additionally, in 2016, the US FDA changed the RRR to all-racemic α-tocopherol ratio of biopotency from 1.36:1 to 2:1 for food-labeling purposes [8].

Even though both sources of α-tocopherol, the naturally sourced RRR- and synthetic all-racemic- are commonly consumed from foods and dietary supplements, natural RRR α-tocopherol is more likely to have the most significant effect on health outcomes. Because of vitamins’ potential health benefits, there is considerable interest in plants with high vitamin E content. There are considerable research efforts to find a suitable/sustainable way to produce tocopherol, especially α-tocopherol. Therefore, soybean callus could possibly be used as a source of α-tocopherol with health benefits for functional foods and nutraceuticals.

Recently, new biotechnological approaches have been introduced to produce a variety of phytochemicals, bioactives, and nutrients from plants and microalgae in vitro cultures [9]. Significantly, the biotechnological production of controlled culture conditions has been greatly explored to obtain phytochemicals and nutrients from calli [10]. Most plant species required both the cytokinins and auxins for maximum callus induction [11]. Furthermore, to produce superior ingredients from callus culture, the callus induction and proliferation depend on the effects of plant growth regulators (PGRs) and their concentrations as well as the specific plant species [12]. For example, in *Sonchus arvensis* L. the combination of 1 mg/L 2.4-dichlorophenoxy acetic acid (2.4-D) and 0.5 mg/L 6-Benzylaminopurine (BAP) was the best treatment, having the shortest induction time and the highest callus number [13]. In *Glossonema varians*, a combination of 2.4-D and α-naphthalene acetic acid (NAA) was found to be most desirable for optimum growth of callus [14]. In *Polyalthia bullata* the picloram was more advantageous for callus induction, while dicamba was the best for callus multiplication [15]. In *Azadirachta indica* the highest callus weight and accumulation of azadirachtin was recorded in green callus induced using thiadiazuron (TDZ) [16].

With regard to soybean, several reports showed that soy callus could be used to produce phytochemicals and nutrients [17,18,19,20,21,22,23]. For example, soybean callus or soybean callus suspension cultures were reported to have isoflavones [24,25,26]. Furthermore, soybean callus was reported to be utilized in increasing the production of tocopherols in soybeans by accelerating the flow of biosynthesis with an increased total tocopherol content [27].

Based on these reports, there is a possibility of using soybean callus as a sustainable source to produce tocopherol. However, despite the potential of soybean callus as a possible source of tocopherols, there is missing information about the possible effects of commonly used herbicide uses on soybean callus production. There is enough evidence that treatment with pesticides and herbicides may change the nutritional quality of food crops, especially the vitamin content [28].

Therefore, in this study, we investigated three aims: the production of soybean calli from soybeans cultivated under three different weed controls (without herbicide and with two variants of herbicide); the determination of the potential effects of weed control methods on tocopherol contents; and the usability of soybean callus as a suitable source of α-tocopherol (α-toco), γ-tocopherol (γ-toco), and δ-tocopherol (δ-toco).

## 2. Results

### 2.1. Callus Culture Initiation

**Figure 1:**
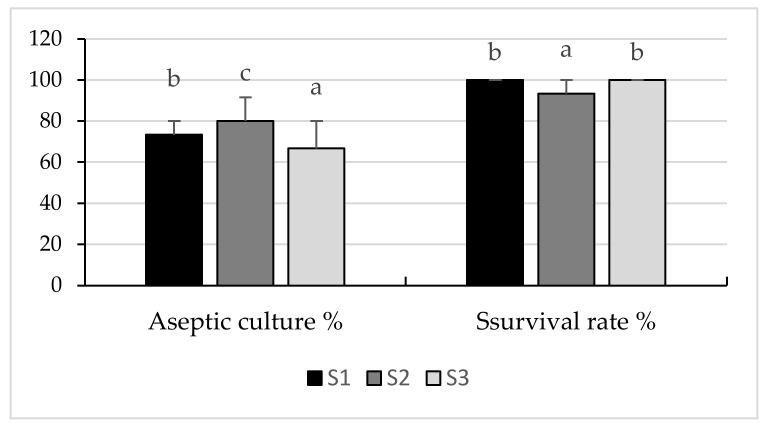
Aseptic culture and survival rate at the initiation of in vitro culture of soybean callus, cv Felix, on Murashige and Skoog 1962 culture medium with 0.1 mg/L 6-Benzylaminopurine + 0.1 mg/L Thidiazuron. S1—conventional soil system: plow + seedbed preparation with rotary harrow + seeding + fertilizer, no herbicide. S2—conventional soil system: plow + seedbed preparation with rotary harrow + seeding + fertilizer + S-metolachlor 960 g/L, imazamox 40 g/L and propaquizafop 100 g/L, with 1.5 L/ha, 0.8 L/ha and 1.5 L/ha. S3—conventional soil system: plow + seedbed preparation with rotary harrow + seeding + fertilizer + dimethenamid 720 g/L, bentazone 480 g/L and fluazifop-P-butyl 150 g/L, with 1.2 L/ha, 2.5 L/ha, and 1.5 L/ha. The values shown are means ± S.E (standard error). Different letters above the bars indicate significant differences according to Tukey’s HSD test (*p* ˂ 0.05).

Aseptic cultures in the initiation phase were obtained by disinfecting the soybeans with a concentration of 20% ACE (5% active chlorine solution) and using a culture medium of MS + 0.1 mg/L BAP + 0.1 mg/L TDZ with values between 66.67–80.00%. Additionally, a survival rate was found to be between 93.33–100% (Figure 1).

### 2.2. Callus Proliferation

**Figure 2:**
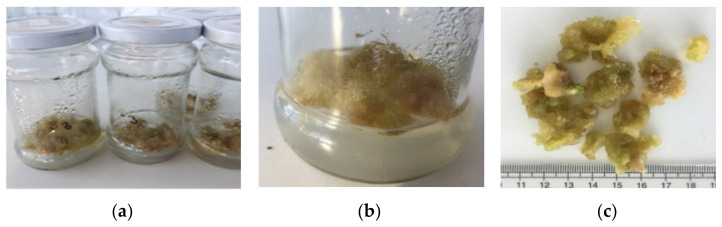

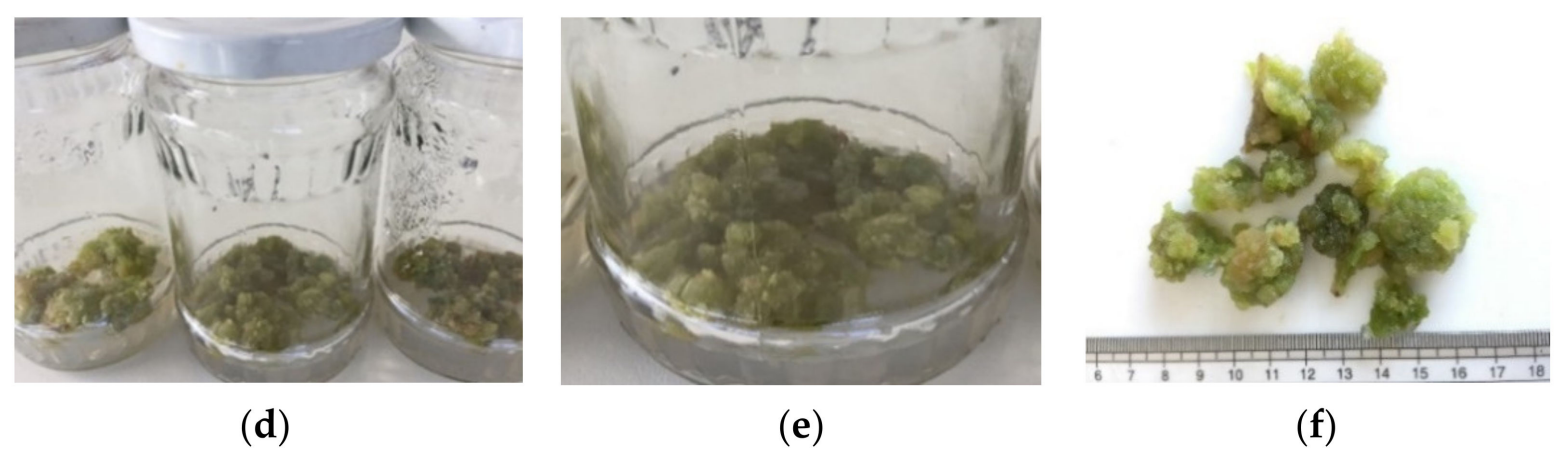
Fresh callus Felix variety (S3—conventional soil system: plow + seedbed preparation with rotary harrow + seeding + fertilizer + dimetenamid 720 g/L, bentazon 480 g/L and fluazifop-P-butil 150 g/L, with 1.2 L/ha, 2.5 L/ha and 1.5 L/ha): (**a**–**c**) Callus obtained on the medium MS + 1.5 mg/L 2.4 D; (**d**–**f**) Callus obtained on the medium MS + 0.1mg/L BAP + 0.1mg/L TZD.

The callus from the MS medium with 1.5 mg/L 2.4D had a yellowish color and a friable consistency, while the callus from the MS medium with 0.1 mg/L BAP + 0.1 mg/L TDZ had a green color (Figure 2).

**Figure 3:**
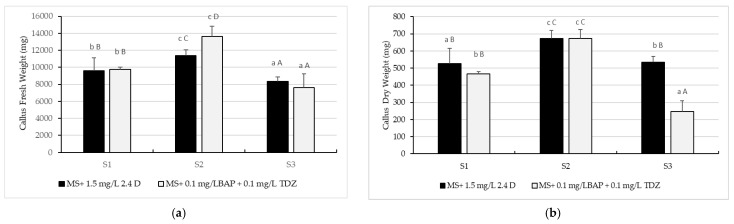
The amount of fresh soybean callus (**a**) and dry (**b**) soybean callus, cv Felix, obtained on Murashige and Skoog 1962 culture medium supplemented with 0.1 mg/L 6-Benzylaminopurine + 0.1 mg/L Thidiazuron and 1.5 mg/L 2.4-Dichlorophenoxy Acetic Acid. S1—conventional soil system: plow + seedbed preparation with rotary harrow + seeding + fertilizer, no herbicide. S2—conventional soil system: plow + seedbed preparation with rotary harrow + seeding + fertilizer + S-metolachlor 960 g/L, imazamox 40 g/L and propaquizafop 100 g/L, with 1.5 L/ha, 0.8 L/ha, and 1.5 L/ha. S3—conventional soil system: plow + seedbed preparation with rotary harrow + seeding + fertilizer + dimethenamid 720 g/L, bentazone 480 g/L and fluazifop-P-butyl 150 g/L, with 1.2 L/ha, 2.5 L/ha, and 1.5 L/ha. The values shown are means ± S.E. Different lowercase letters indicate significant differences between the means within the same culture medium, while capital letters indicate significant differences between the means of the three weed control systems according to Tukey’s HSD test (*p* ˂ 0.05).

Our data show that the combination of the two plant growth regulators (PGRs; see Materials and Methods for details) did not significantly affect callus production (*p* ˂ 0.05) (Figure 3a). However, there were statistically significant differences between the amount of fresh callus obtained from soybeans from the three different weed control systems. The highest amount of fresh callus came from soybeans treated with the second weed control on both variants of culture medium; 13,652.4 ± 1177.621 mg on MS culture medium supplemented with + 0.1 mg/LBAP + 0.1 mg/L Thidiazuron (TDZ) and 11,405.3 ± 671.67 mg on culture medium supplemented MS with 1.5 mg/L 2.4 D. The lowest amount of fresh callus was obtained in the case of soybeans treated with the third weed control; 7606.625 ± 1614.714 mg per culture medium MS supplemented with 0.1 mg/LBAP + 0.1 mg/L TDZ and 8361.875 ± 535.1 mg per medium of culture MS supplemented with 1.5 mg/L 2.4 D.

The highest dry weight (DW) was observed in soybeans cultivated in the soil tillage system with the second weed control on both variants of culture medium; 673.72 ± 52.21 mg on MS culture medium supplemented with + 0.1 mg/L BAP + 0.1 mg/L TDZ and 671.67 ± 47.12 mg on MS culture medium supplemented with 1.5 mg/L 2.4 D. The lowest amount of fresh callus was obtained from soybeans from the third weed control; 7606.62 ± 1614.71 mg on MS culture medium supplemented with 0.1 mg/LBAP + 0.1 mg/L TDZ and 8361.88 ± 535.1 mg on culture medium supplemented with 1.5 mg/L 2.4 D (Figure 2).

The lowest FW was observed in the case of soybeans cultivated in the tillage system with the third weed control on the MS culture medium supplemented with 0.1 mg/LBAP + 0.1 mg/L TDZ; 247.65 ± 62.53 mg (Figure 3b).

The highest growth index (Gi) was also observed in soybeans cultivated in the soil tillage system with the second weed control on both variants of culture media; 1265.23 ± 117.76% on MS culture medium supplemented with 0.1 mg/L BAP + 0.1 mg/L TDZ and 1040.53 ± 110.56% on MS culture medium supplemented with 1.5 mg/L 2.4 D.

**Figure 4:**
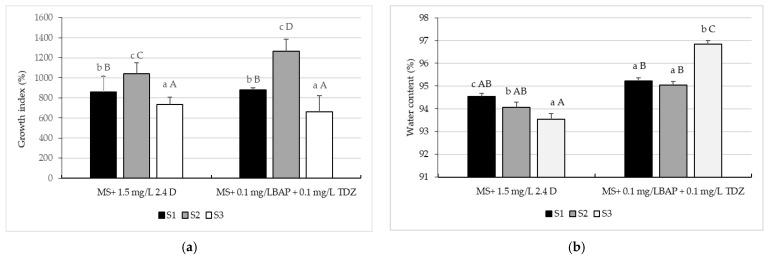
Growth index (**a**) and water content (**b**) for soybean callus, cv Felix, obtained on Murashige and Skoog 1962 culture medium supplemented with 0.1 mg/L 6-Benzylaminopurine + 0.1 mg/L Thidiazuron and 1.5 mg/L 2.4-Dichlorophenoxy Acetic Acid. S1—conventional soil system: plow + seedbed preparation with rotary harrow + seeding + fertilizer, no herbicide. S2—conventional soil system: plow + seedbed preparation with rotary harrow + seeding + fertilizer + S-metolachlor 960 g/L, imazamox 40 g/L and propaquizafop 100 g/L, with 1.5 L/ha, 0.8 L/ha, and 1.5 L/ha. S3—conventional tillage system: plow + seedbed preparation with rotary harrow + seeding + fertilizer + dimethenamid 720 g/L, bentazone 480 g/L and fluazifop-P-butyl 150 g/L, with 1.2 L/ha, 2.5 L/ha, and 1.5 L/ha. The values shown are means ± S.E. Different lowercase letters indicate significant differences between the means of the same culture medium, while capital letters indicate significant differences between the means of the two-culture medium according to Tukey’s HSD test (*p* ˂ 0.05).

The lowest Gi was detected in callus obtained from soybeans cultivated in the tillage system with the third weed control on both culture medium (statistically insignificant difference); 660.66 ± 161.47% in the case of callus obtained on the medium of culture MS supplemented with 0.1 mg/LBAP + 0.1 mg/L TDZ and 736.18 ± 71.71% on culture medium MS supplemented with 1.5 mg/L 2.4 D (Figure 4a).

The highest and the lowest callus water content (CWC) was found in the callus obtained from soybeans cultivated using the tillage system with the third weed control on both culture media, respectively 93.55% on the MS culture medium supplemented with 1.5 mg/L 2.4 D and 96.84% on MS culture medium supplemented with 0.1 mg/L BAP + 0.1 mg/L TDZ. In the case of the other variants, there were no statistically significant differences in CWC (Figure 4b).

### 2.3. Tocopherol Content

**Figure 5:**
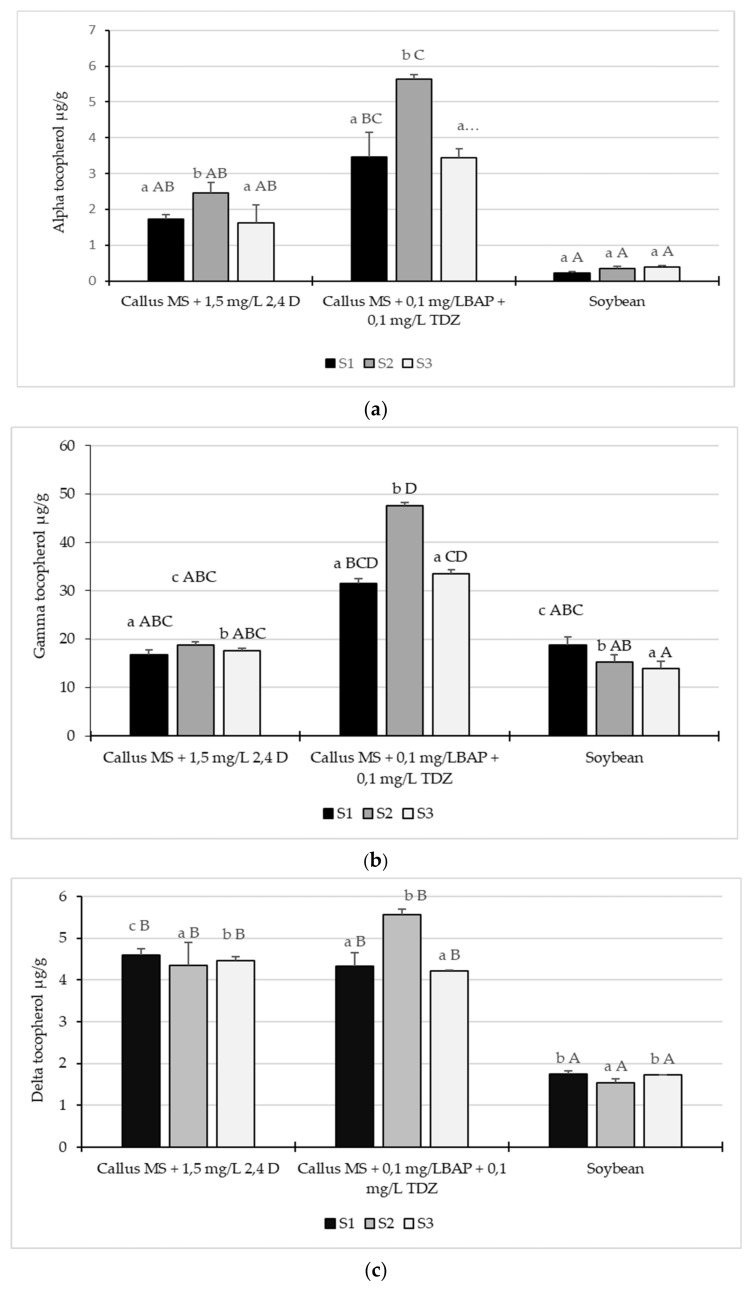
Content of tocopherols in soybean seeds and soybean callus cv Felix: (**a**) α tocopherol; (**b**) γ tocopherol; and (**c**) δ tocopherol. S1—conventional soil system: plow + seedbed preparation with rotary harrow + seeding + fertilizer, no herbicide. S2—conventional soil system: plow + seedbed preparation with rotary harrow + seeding + fertilizer + S-metolachlor 960 g/L, imazamox 40 g/L and propaquizafop 100 g/L, with 1.5 L/ha, 0.8 L/ha, and 1.5 L/ha. S3—conventional soil system: plow + seedbed preparation with rotary harrow + seeding + fertilizer + dimethenamide 720 g/L, bentazone 480 g/L and fluazi-fop-P-butyl 150 g/L, with 1.2 L/ha, 2.5 L/ha, and 1.5 L/ha. The values shown are means ± S.E. Different lowercase letters indicate significant differences between the means of the three weed control systems within every medium and soybean seeds. Capital letters indicate significant differences between the means of all analyzed callus samples and the soybean seeds according to Tukey’s HSD test (*p* ˂ 0.05).

The data showed that α-toco was significantly lower (*p* ˂ 0.05) in soybeans cultivated in all three weed control systems than the callus grown in culture medium MS + 0.1 mg/L BAP + 0.1 mg/L TDZ. However, there were no statistically significant differences in the amount of tocopherols between the callus soybeans cultivated in MS + 1.5 mg/L 2.4 D medium (Figure 5a).

The highest α-toco content was 5.63 ± 0.12 µg/g in callus obtained from soybeans cultivated using the second weed control system. The lowest tocopherol content was 0.23 ± 0.04 µg/g in soybeans grown using the conventional tillage system without herbicide (first weed control) (Figure 5a).

In both soybeans and callus, the content of γ-toco was higher than the α- and δ-toco in all samples collected from the three weed control systems. Similar to α-toco, the highest γ-toco content was in the callus obtained from soybeans cultivated in the second weed tillage system; 47.57 ± 0.72 µg/g. These results showed a statistically significant difference in the amounts of tocopherol (*p* ˂ 0.05) between the callus samples obtained from the first and third weed control groups. The lowest γ-toco content was 13.86 ± 1.60 µg/g in soybeans cultivated using the conventional tillage system with the third weed control (Figure 5b).

However, there was no statistically significant difference in the amount of tocopherol γ between the soybeans in all three weed control systems and callus cultivated in culture medium MS + 1.5 mg/L 2.4 D, (*p* ˂ 0.05) (Figure 5b).

Regarding the content of δ tocopherol, the data showed no statistically significant difference (*p* ˂ 0.05) between the callus samples cultivated in the two-culture medium, regardless of the soy tillage system. However, the δ tocopherol content in callus was found to be between 4.21 ± 0.10 µg/g and 5.57 ± 0.13 µg/g, which were significantly higher than those of soybeans (between 1.54 ± 0.02 µg/g; I 1.74 ± 0.01 µg/g) (Figure 5c).

## 3. Discussion

Different types of explants can be used to initiate in vitro soybean callus cultures such as immature embryos at different developmental stages, with lengths from 0.5 to 10 mm. [29]; shoot tips and cotyledonary nodal segments [30]; various explants derived from 10-day-old seedlings of soybeans such as whole cotyledon, cotyledonary node, hypocotyl, and root [31]; mature cotyledons and embryos [22]. To increase callus production, Kosturkova and colleagues initiated the in vitro culture of cotyledons and cotyledonary nodes in five different soybean varieties [32]. Since our callus was produced to analyze the tocopherol content and compared it with the one in soybean, we used half of cotyledon and hypocotyl for initiation.

Culture medium MS and cytokinin BA has been frequently used in in vitro soybean cultures [33]. Furthermore, the MS medium, with the combination of cytokinins BAP (0.1 mg/L) and TDZ (0.1 mg/L), has been proved to be an efficient medium for initiating in vitro culture of soybean callus. Using this medium, sterilized soybean treated for 20 min with a concentration of 20% ACE (5% active chlorine) were initiated between 66.67–80%, similar to the results reported by Phat and colleagues [31].

Among the PGRs used in callus production, especially for somatic embryogenesis and plant regeneration in soy, 2.4D has been used in various concentrations, between 3–206 μM [22,23]. In our study, for the production of callus, along with the medium used to initiate the callus culture (MS + 0.1 mg/L BAP + 0.1 mg/L TDZ), we also used the MS + 1.5 mg/L 2.4D. Since our callus was produced as a possible source of tocopherols, the aim was to develop a callus growth protocol that would contain as few PGRs as possible. In contrast to our method, Kumari et al. 2006 [34] used the MS culture medium supplemented with much higher concentrations of PGRs such as BA—2.22 μM with 2.4-D—4.53, 26.6, 45.2, 90.4, 135.6, 180.8, and 226.0 μM for embryogenic callus induction, and the highest frequency (92.9%) was obtained on the supplemented medium with 180.8 µM 2.4-D and 2.22 µM BA (1 mg 2.4 D = 4.55 μM and 1 mg BA = 4.44 μM).

Using the culture medium MS supplemented with 0.1 mg/L BAP + 0.1 mg/L TDZ, a significantly higher amount of fresh callus was obtained than using the medium supplemented with 1.5 mg/L 2.4 D in soybeans cultivated in the conventional system of tillage with second weed control. Additionally, in soybeans obtained with second weed control, on the same culture medium, Gi showed significant differences compared to Gi obtained on the second culture medium.

Based on these data, in this study, four Bulgarian soybean varieties were used in the production of pharmaceuticals and other valuable substances using the basal MS medium with three combinations of PGRs: 1 mg/L kinetin and 0.5 mg/L 2.4-D, 0.5 mg/L kinetin and 1 mg/L 2.4D, 0.1 mg/L kinetin and 0.5 mg/L 2.4D obtaining a callus growth rate between 1.2 and 9.1, depending on the variety, explant, and culture medium [32].

To our best knowledge, this is the first study to regenerate soybean callus from crops with different weed control variants. The data show that the use of herbicides in soybean culture may have an impact on the quantity and quality of soy callus produced in vitro.

For years, soybean seeds have been used as an important source of dietary tocopherols. However, soybean callus has not been explored much as a possible source of tocopherols. Callus culture and suspension-cultured cells are an essential source of tocopherols for several species: *Chenopodium quinoa* [35], *Argania Spinosa* [36], *Helianthus annuus* [37,38], *Carthamus tinctorius* [39], or *Daucus carota* [40].

Our data showed that, in both callus and soybeans, from all three weed control variants, γ-tocopherol was present in a higher amount than α- and δ-tocopherols (Figure 5). For example, γ-tocopherol was 81% in callus compared with 88% in soybean, α-tocopherol was 10% in callus compared with 2% in soybean, and δ-tocopherols was 9% in callus compared with 10% in soybean (second weed control system). Similarly, other results show that γ-tocopherol is reported to comprise about 50–60% of total tocols, while α-tocopherol comprises only 5–10% in soybeans [41,42].

Even though there is no study to compare the content of tocopherols in soybean callus and soybean seeds, there are some data for other plants. For example, in quinoa callus (culture medium MS + 2mg/L 2.4D + 0.05 mg/L kinetine) and suspension-cultured cells from different plant parts, the amount of total tocopherol was higher than the quinoa seeds [35].

Regarding the content of tocopherols in soybean seeds, it is known to be affected by environmental factors, agronomic practices such as the air temperature or soil moisture, weather, storage, soil management system, and field year [1,43]. For example, Britz and Kremer [44] reported that weather or climate could significantly affect seed tocopherols. Seguin et al. [45] found that seeding rate, row spacing, and planting dates could affect the tocopherol concentration in soybeans. Interestingly, Ujiie et al. [46] investigated the genetic variability of tocopherol in soybeans and found that one of three soybean varieties with a higher α-tocopherol content is the Romanian variety Dobrogeance. Even though our Romanian Felix variety has a lower α-tocopherol content, the callus has a much higher amount of α-tocopherol (5.63 µg/g α-toco in callus compared with 0.35µg/g in soybean seeds). Knowing the importance of α-tocopherol in our health, an efficient to produce tocopherol has been explored for years. However, the efforts to increase the tocopherol content of soybean seeds still have only limited results, although the transgenic approach may be more successful.

Altogether, the results of our study show that *Glicine max* L. Merr has proven to be a suitable species that deserves further investigation to establish an efficient callus production protocol as a source of tocopherols.

## 4. Materials and Methods

### 4.1. Plant Material

Soybeans used in this study were a high-grade Felix variety (*Glycine max* L. Merr.) They were obtained from the experimental field of Agricultural Research and Development Station in Turda, Romania, through the conventional tillage system [47] in the following experimental variants:

S1—conventional soil system: plow + seedbed preparation with rotary harrow + seeding + fertilizer, no herbicide—first weed control.

S2—conventional soil system: plow + seedbed preparation with rotary harrow + seeding + fertilizer + S-metolachlor 960 g/L, imazamox 40 g/L and propaquizafop 100 g/L, with 1.5 L/ha, 0.8 L/ha, and 1.5 L/ha—second weed control.

S3—conventional soil system: plow + seedbed preparation with rotary harrow + seeding + fertilizer + dimethenamid 720 g/L, bentazone 480 g/L and fluazifop-P-butyl 150 g/L, with 1.2 L/ha, 2.5 L/ha, and 1.5 L/ha—third weed control.

### 4.2. Callus Initiation and Subculture

The soybeans were washed for 10 min under running water and then for another 10 min in sterile distilled water with a drop of Tween. They were then rinsed in sterile distilled water until Tween residues were removed and sterilized in a 20% ACE solution (5% active chlorine) for 20 min. The sterilized soybeans were kept for six hours in sterilized deionized water. For the initiation of in vitro callus culture was used only the part of the cotyledon that contained the hypocotyl. They were placed in test tubes, on the culture medium Murashige and Skoog (1962) [48] (MS) supplemented with 30 g sugar (Coronița AGRANA Romania), 0.1 mg/L 6-benzylaminopurine (BAP) + 0.1 mg/L thidiazuron (TDZ), and gelled with 5 g/L plant agar. All the materials were obtained from Duchefa, (Haarlem, The Netherlands).

Six weeks after inoculation, the percentage of uncontaminated cultures and the sur-vival rate of aseptic cultures were determined.

The callus obtained in the whole phase was sub-cultured three times on the MS cul-ture medium supplemented with the following plant growth regulators (PGRs):

V1—MS + 1.5 mg/L 2.4-Dichlorophenoxy Acetic Acid (2.4D)

V2—MS + 0.1 mg/L BAP + 0.1 mg/L TDZ

For the callus proliferation phase, we used 370 mL jars with a lid that had a 50 mL filter medium/jar (4 jars/variant/3 repetitions) were used. 1 g of callus/jar was inoculated. The duration of a subculture was 6 weeks.

The in vitro cultures were maintained at 16/8 h light/dark photoperiod at 32.4 μmol·m^−2^s^−1^ light intensity and 23 ± 3 °C in a growth room.

After each subculture, fresh callus weight (FW) (mg) was determined, and then the callus was dried under laboratory conditions until a constant weight was obtained and callus dry weight (DW) (mg) was determined.

The callus water content (CWC) (%) was calculated using the following formula [49]

CWC (%) = Fresh weight − Dry weight/Dry weight × 100

The biomass increase on a fresh weight basis is expressed as the percent increment over the initial inoculum (growth index-Gi) that was calculated as follows [50]

Gi = (Final mass − Initial mass)/Initial mass × 100

The analysis of the tocopherol content was done on the dry callus obtained from the third subculture.

### 4.3. Extraction and Analysis of Tocopherols

γ- and δ-tocopherols are found in higher concentrations in soybeans, accounting for 66% and 29% of total tocopherols, respectively [51]. α-toco accounts for less than 10%, and β-tocopherol accounts for less than 1% of the composition of tocopherols in soybeans [52]. We were not able to identify the β-tocopherol, so its analysis is not included in this study.

For the tocopherol’s extraction, the soybeans and the dried soybean callus were finely ground, and one gram was used for each extraction. The extractions were performed in triplicate, in the dark, at room temperature.

For each sample, a sterile conical tube (15 mL, 17 × 120 mm) was prepared with 0.25 g ascorbic acid over which the soybean sample was added. 7.3 mL of the saponification solution was added to each sample and mixed until the ascorbic acid was completely dissolved. The saponification solution was prepared from 27.5 g of potassium hydroxide (KOH), 75 mL of molecular water, and 137.5 mL of ethyl alcohol (EtOH) (Sigma-Aldrich, St. Louis, MO, USA).

The soybean tubes and saponification solution were placed in a hot water bath at 80° C for 15 min, and then the tubes were removed and stirred for five minutes. The operation was repeated three times in 15 min of stirring and 30 min in the hot bath. To stop the reaction, the tubes were placed on ice for 10 min. We added 4 mL of hexane to each tube, and the solution was stirred for two minutes. The tubes were centrifuged for 10 min at 3000 rpm. The supernatant was transferred to another tube with 3 ml of cold molecular water. The solution was stirred for one minute and centrifuged at 3000 rpm for 10 min, after which the stirring–centrifugation operation was repeated. The solution was filtered through a 25 mm (0.2 µm Nylon) non-sterile syringe filter and transferred to a dark glass container to protect the solution from light. A volume of 10 µL from each sample was injected into the HPLC.

HPLC analysis was performed with Waters e2695 separation module equipped with Waters 996 detector. The reverse-phase column (Waters-WAT086344-Nova-Pak C18 4 um, 3.9 × 150 mn, Waters, Dublin, Ireland) was used, and the separation was made with an isocratic elution, with a mobile phase containing Acetonitrile/Methanol in a proportion of 75:25 v/v. The samples were separated using a gradient condition; buffer A (50 mM NaH2PO4, pH 4.3) and buffer B (acetonitrile × methanol 3:1). The temperature was maintained at 20 °C at a rate of 0.8 mL/min for 60 min. Tocopherol was detected between 250 and 300 nmUV.

For the quantitative analysis, standard tocopherols were used at different concentrations to allow the construction of a standard curve with five different concentrations. δ-, γ-, and α-tocopherol standards (Sigma Aldrich, St. Louis, MO, USA) were used to prepare calibration curves, and the individual tocopherols were quantified.

### 4.4. Statistical Analysis

To analyze the data, ANOVA analysis was performed first to check the differences among the means. When the null hypothesis was rejected, Tukey’s HSD test (*p* ˂ 0.05) was performed to determine the means that were significantly different from each other. The values shown are means ± SE (standard error).

## 5. Conclusions

Attributable to the health-promoting benefits of tocopherols, many approaches have been emerged to increase their production thru various methods, especially the production of α-tocopherol, which exhibits the highest biological vitamin E activity. The uses of in vitro cultures are bio-sustainable and cost-effective. Our study evaluated the possibility of using soybean callus to mass-produce tocopherols. MS medium supplemented with 0.1 mg/L BAP + 0.1 mg/L TDZ was the suitable medium for soybean callus production. The results are encouraged, as all the analyzed tocopherols are present in much higher content in soy callus than the soybean seeds (5.63 µg/g compared with the 0.35 α-toco in soybean, 47.57 µg/g compared with 18.71 µg/g γ-toco or, 5.56 µg/g compared with 1.74 µg/g β-tocopherol). Furthermore, to our best knowledge, this is the first time when seeds from the conventional tillage system were used for the initiation of the soy callus with three different weed controls (without herbicide and with two herbicide treatments). Our data showed that in both callus and soybeans from all three weed control variants, γ-tocopherol was present in a higher amount than α- and δ-tocopherols. Notably, this is the first report confirming that the biologically active α-tocopherol, is found in higher concentrations of soy callus than soybean. In soybeans obtained in second weed control system α-tocopherol was 10% in callus compared with 2% in soybean, γ-tocopherol was 81% in callus compared with 88% in soybean, and δ-tocopherols was 9% in callus compared with 10% in soybean. It has been found that herbicides used in soybean culture can influence both the amount of callus obtained in vitro and its tocopherol content. As there are many advantages of in-vitro or cell cultures compared with the conventional whole plant cultivation, without a doubt, the exploration of soybean for the large-scale production of α-toco should be taken into consideration. In addition, further studies are needed to investigate whether there are changes in callus tocopherol content after several subcultures.

## Data Availability

The data presented in this study are available in the article.
